# T-Channel Field Effect Transistor with Three Input Terminals (Ti-TcFET)

**DOI:** 10.3390/mi11010064

**Published:** 2020-01-07

**Authors:** Zeqi Chen, Jianping Hu, Hao Ye, Zhufei Chu

**Affiliations:** Faculty of Information Science and Technology, Ningbo University, Ningbo 315211, China; 1711082003@nbu.edu.cn (Z.C.); 1801082027@nbu.edu.cn (H.Y.); chuzhufei@nbu.edu.cn (Z.C.)

**Keywords:** new device, three-input transistor, T-channel, compact circuit style

## Abstract

In this paper, a novel T-channel field effect transistor with three input terminals (Ti-TcFET) is proposed. The channel of a Ti-TcFET consists of horizontal and vertical sections. The top gate is above the horizontal channel, while the front gate and back gate are on either side of the vertical channel. The T-shaped channel structure increases the coupling area between the top gate and the front and back gates, which improves the ability of the gate electrodes to control the channel. What’s more, it makes the top gate have almost the same control ability for the channel as the front gate and the back gate. This unique structure design brings a unique function in that the device is turned on only when two or three inputs are activated. Silvaco technology computer-aided design (TCAD) simulations are used to verify the current characteristics of the proposed Ti-TcFET. The current characteristics of the device are theoretically analyzed, and the results show that the theoretical analysis agrees with the TCAD simulation results. The proposed Ti-TcFET devices with three input terminals can be used to simplify the complex circuits in a compact style with reduced counts of transistors compared with the traditional complementary metal–oxide–semiconductor/ fin field-effect transistors (CMOS/FinFETs) with a single input terminal and thus provides a new idea for future circuit designs.

## 1. Introduction

Because of short-channel effects, the size of metal-oxide-semiconductor (MOS) devices is seriously restricted. In order to continue Moore’s law, many new device structures have been proposed, such as silicon-on-insulator metal-oxide-semiconductor field-effect-transistors (SOI MOSFETs) with a single-gate structure, fin field-effect transistor (FinFETs) with a double-gate structure [1], and tri-gate field effect transistors (FETs) [2], Ω-gate FETs [3], and Gate-All-Around (GAA)FETs with a multi-gate structure. Among these devices, FinFET has been widely used in chip fabrication since it can considerably improve the ability of the gate electrode to control the channel and thus suppress the short-channel effects.

Most of the multi-gate devices mentioned above have only one input terminal. However, previous studies have shown that designing a circuit with devices with multiple input terminals is more flexible and efficient than using single-input ones [4,5]. The two-input low-threshold FinFET device proposed in the literature [6,7,8,9,10] is equivalent to two parallel transistors, while the two-input high-threshold FinFET device is equivalent to two series transistors. Therefore, the circuit can be simplified to reduce the transistor count by using two-input low-threshold and high-threshold FinFETs, thus reducing power consumption and the chip area. If a device has more input terminals, it is possible to achieve more flexible and efficient circuit designs.

This paper proposes a novel T-channel field effect transistor with three input terminals (Ti-TcFET). The invented T-type channel structure allows the device to have three independent input gates: the top gate, front gate, and back gate. Because of the device structure with a T-type channel, the coupling areas among the top gate and the front and back gates are increased, which increases the control capability of the gates to the channel. This unique structure design brings a unique function in that the device is turned on only when two or three inputs are activated. Compared with traditional FinFETs with a single input terminal, the proposed Ti-TcFET devices with three input terminals can provide more flexible circuit realizations in a compact style. The proposed Ti-TcFET devices can be fabricated by adding only a small number of process steps on the basis of the current mainstream FinFET process. 

This paper is organized as follows. In Section 2, the structure of the proposed device is introduced, and its device parameters are presented. The key processing steps of Ti-TcFET devices are also included in Section 2. In Section 3, the current characteristics of the device are theoretically analyzed, and Silvaco technology computer-aided design (TCAD) simulations are used to verify the accuracy of theoretical analysis. In Section 3, we illustrate how to carry out performance optimization for the proposed Ti-TcFET devices, and device performances are analyzed and evaluated in terms of turn-on and turn-off currents and switching current ratio. The compact circuits based on Ti-TcFET devices are also included in Section 3. We show that Ti-TcFET devices can be used to simplify complex circuits in a compact style with reduced transistor counts compared with the traditional complementary metal–oxide–semiconductor/ fin field-effect transistors (CMOS/FinFETs) with a single input terminal. Finally, the work of this paper is summarized in the last section.

## 2. Device Structure and Description

This section takes an N-type Ti-TcFET as an example to present the structure and parameters of the proposed device. We also give the fabrication process of Ti-TcFET devices in this section.

### 2.1. The Structure of the Ti-TcFET

**Figure 1:**
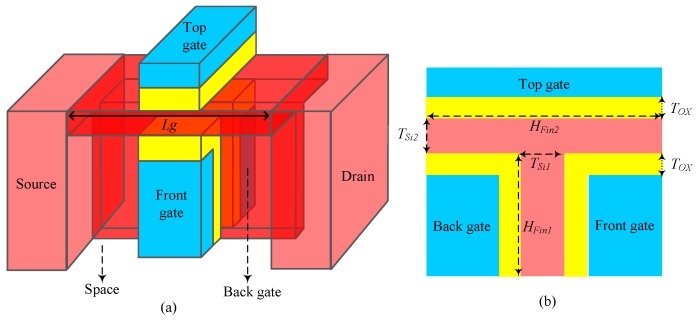
Structure of the N-type T-channel field effect transistor with three input terminals (Ti-TcFET) device: (**a**) 3D diagram, and (**b**) cross-sectional view.

Figure 1a shows a 3D diagram of the N-type Ti-TcFET, while Figure 1b is a cross-sectional diagram of the N-type Ti-TcFET. As seen in Figure 1a, the device has three independent input gates, termed the top gate, front gate, and back gate. From Figure 1b, we can see that the T-type channel of the Ti-TcFET is divided into horizontal and vertical sections. *H_Fin1_* and *T_Si1_* are the fin height and thickness of the vertical channel, respectively, while *H_Fin2_* and *T_Si2_* are the fin height and thickness of the horizontal channel, respectively. By adjusting the fin height *H_Fin2_* of the horizontal channel, the contact area between the top gate and the horizontal channel can be changed, thus changing the coupling strength between the top gate and the front and back gates. The gate-to-channel control capability can be varied by adjusting the gate work function and the thickness *T_ox_* of the high-K dielectric hafnium(IV) oxide (HfO_2_).

In this work, SiO_2_ is used as the substrate material for the device. The channel material is silicon, and the gate oxide material employs high-K dielectric HfO_2_. The device parameters of the Ti-TcFET are shown in Table 1. The optimum values of *H_Fin1_ T_Si1_*, *H_Fin2_*, *T_Si2_*, *T_OX_* (HfO_2_ thickness), and *L_g_* (channel length) are listed in Table 1. The doping concentrations *N*_drain_ and *N*_source_ of the source and drain regions are 2 × 10^20^ cm^−3^, and the channel doping concentration *N*_channel_ is 1 × 10^16^ cm^−3^. The gate work function *Φ_m_* of the N-type Ti-TcFET is set to 4.95 eV, while the gate work function of the P-type Ti-TcFET is selected to be 4.55 eV.

### 2.2. Key Processing Steps for the Ti-TcFET Device

**Figure 2:**
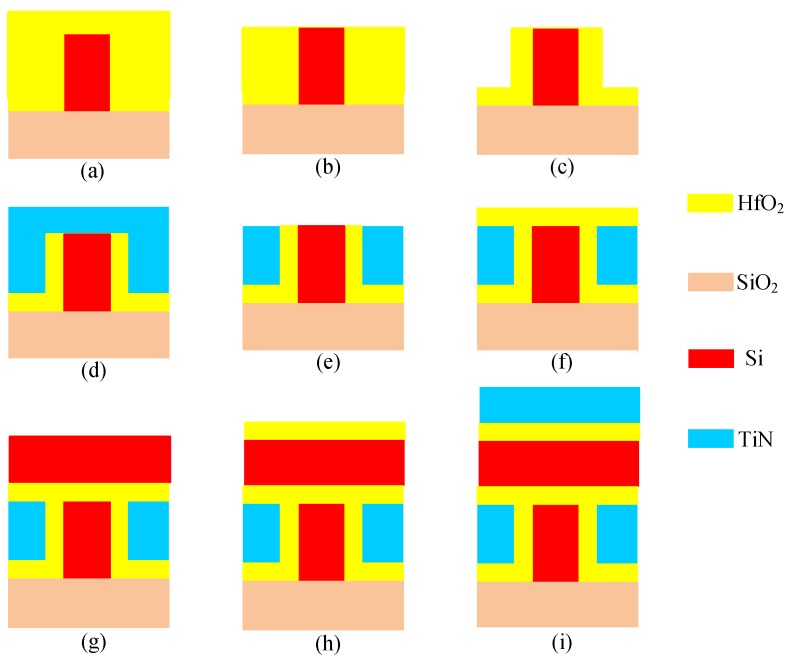
Key process steps of the Ti-TcFET device. (**a**) Silicon-on-insulator (SOI) layers were etched down to the buried oxide layer to produce the bodies of the devices; (**b**) chemical mechanical polishing (CMP) processing was used to remove the extra part of gate oxide above the top of the fin; (**c**) the extra part of gate oxide was etched back; (**d**) a Si_3_N_4_ gate electrode mask was deposited and patterned; (**e**) the gate pattern was etched into the Si_3_N_4_ and through the TiN to form the gate electrodes; (**f**) the high-K dielectric was deposited by using atomic layer deposition (ALD) processing; (**g**) a suitable horizontal channel structure was established by using smart-cut processing; (**h**) the high-K dielectric layer was deposited by using ALD processing; (**i**) the gate pattern was etched into the silicon and through the TiN to form the top gate electrodes.

On the basis of a traditional SOI-independent FinFET process [11], Ti-TcFET devices can be fabricated by adding a few processing steps. A key fabrication process is shown in Figure 2.

A SiO_2_ film was firstly grown as a mask for the silicon fin etching, and then the hard-mask and SOI layers were etched down to the buried oxide layer to produce the bodies of the devices, as shown in Figure 2a [11]. A high-K dielectric layer was deposited by using atomic layer deposition (ALD) processing. Next, chemical mechanical polishing (CMP) processing was used to remove the extra part of gate oxide above the top of the fin, as shown in Figure 2b. The extra part of gate oxide was etched back, and only the required part on the two sides of the fin remained, as shown in Figure 2c [11]. Thereafter, a Si_3_N_4_ gate electrode mask was deposited and patterned, as shown in Figure 2d. The gate pattern was etched into the Si_3_N_4_ and through the TiN to form the gate electrodes, as shown in Figure 2e. The high-K dielectric was deposited by using ALD processing, as shown in Figure 2f. In order to reduce the difficulty of the process, the horizontal channel and vertical channel were separated by HfO_2_, as shown in Figure 2g. Figure 2g shows how a suitable horizontal channel structure was established by using smart-cut processing. In order to establish the gate oxide layer of the top gate, the high-K dielectric layer was deposited by using ALD processing, as shown in Figure 2h. A Si_3_N_4_ gate electrode mask was deposited and patterned, and finally, the gate pattern was etched into the silicon and through the TiN to form the top gate electrodes, as shown in Figure 2i.

## 3. Results and Discussion

In this section, the current characteristics of the device are theoretically analyzed by modeling the threshold voltage of Ti-TcFETs, and then Silvaco TCAD simulations are used to verify the accuracy of the theoretical analysis. The Lombardi constant voltage and temperature (CVT), Fermi–Dirac carrier statistics (FERMIDIRAC), Shockley–Read–Hall (SRH) models, and the Bohm quantum potential (BQP) models were considered in these TCAD simulations [12]. The performance optimizations for the proposed Ti-TcFET devices were carried out by selecting the channel thickness, gate oxide thickness, and gate work function, and device performances were evaluated in terms of turn-on and turn-off currents and switching current ratio. The several basic logic cells such as the “majority-not” [13,14], NOT-AND (NAND), and NOT-OR (NOR) logic cells, and the full adder realized by using the proposed Ti-TcFET devices are illustrated, showing that Ti-TcFET devices can be used to simplify complex circuits in a compact style with reduced transistor counts.

### 3.1. The Threshold Voltage of Ti-TcFET Devices

The Ti-TcFET device has three inputs. The threshold voltage of any gate is affected by the bias voltage of the other two gates because of the coupling effect among the three gates. In other words, the threshold voltage of a gate is a function of the voltages of the other two gates. Taking the threshold voltage of the top gate of the N-type Ti-TcFET as an example, the relationship between the threshold voltage and other gate voltages (front gate and back gates) is analyzed.

The relationship between the threshold voltage of the top gate and the bias voltages of the front gate and back gate can be measured by introducing the coupling coefficients *γ_top-gate_* and *γ_top-back_* [15,16], as shown in Equations (1) and (2).

(1) $$r_{top\text{-}front}=\frac{\Delta V_{THt}}{\Delta V_{front\text{-}gate}}=\frac{C_{si}\cdot C_{oxf}}{C_{oxt}\cdot \left(3C_{oxf}+2C_{si}\right)}≅\frac{T_{oxf}}{2T_{oxf}+6.3T_{si}},$$

(2) $$r_{top\text{-}back}=\frac{\Delta V_{THt}}{\Delta V_{back\text{-}gate}}=\frac{C_{si}\cdot C_{oxb}}{C_{oxt}\cdot \left(3C_{oxb}+2C_{si}\right)}≅\frac{T_{oxb}}{2T_{oxb}+6.3T_{si}},$$

where *V_THt_* is the threshold voltage of the top gate, *V_front-gate_* and *V_back-gate_* are the voltages of the front gate and back gate, respectively, *C_oxf_*, *C_oxb_,* and *C_oxt_* are the oxide capacitance of the front gate, back gate, and top gate, respectively, *C_si_* and *T_si_* are the body capacitance and thickness of the channel, respectively, and *T_oxf_* and *T_oxb_* are e thickness of the front and back gates, respectively.

TCAD simulation show that the threshold voltage *V_THt_* of the top gate is not completely linear to the bias voltages of the front and back gates. After considering the secondary effect, the threshold voltage *V_THt_* of the top gate can be written as Equation (3).

(3) $$\begin{array}{l} V_{THt}=V_{TH0}-r_{top\text{-}\mathrm{front}}\cdot V_{front\text{-}gate}-r_{top\text{-}back}\cdot V_{back\text{-}gate}-\alpha \cdot \left(V_{front\text{-}gate}^{2}+V_{back\text{-}gate}^{2}\right)\\ -\beta \cdot V_{front\text{-}gate}\cdot V_{back\text{-}gate} \end{array}$$

where *V_TH0_* is the threshold voltage of the top gate when both *V_front-gate_* and *V_back-gate_* are at 0 V, and *α* and *β* are the fitting parameters.

**Figure 3:**
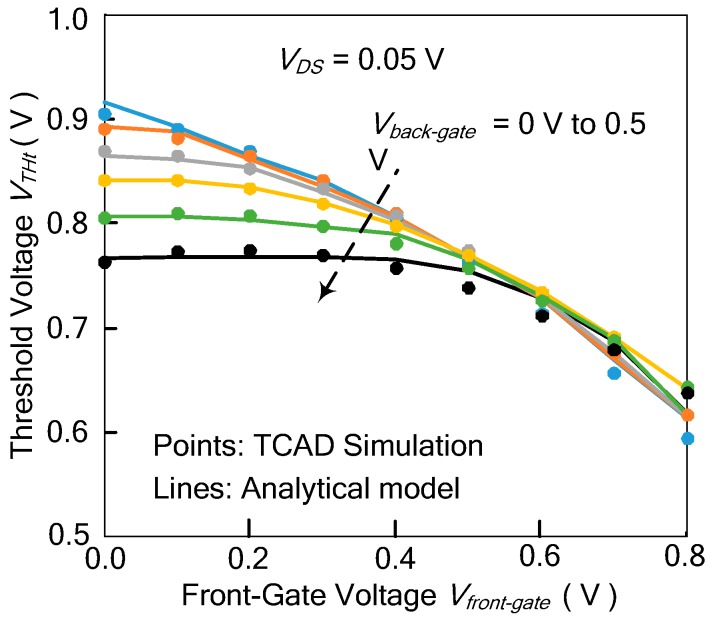
Comparison between calculated and simulated threshold voltages of the top gate versus the front gate in different back gate voltages at *V_DS_* = 50 mV.

The threshold voltage of the top gate versus the voltages of the front gate and the back gate is shown in Figure 3. In Figure 3, the points are the threshold voltages of the Ti-TcFET device obtained by the TCAD simulations, while the lines are the theoretical calculation results obtained by Equation (3) in different voltages of the front and back gates. The results show that the theoretical formula of the threshold voltage agrees with the TCAD simulation results.

As the size of nanoscale devices decreases, quantum mechanical effects will begin to affect device performance. The threshold voltage drift Δ*V_TH_* caused by the quantum mechanical effect can be written as follows [17]:

(4) $$\Delta V_{TH}=\frac{S}{v_{T}\ln10}\Delta \Psi ,$$

where *v_T_* = k*T*/*q* is the thermal voltage—where k is Boltz constant, *T* is the thermodynamic temperature, and *q* is the electronic charge quantity—and *S* is the subthreshold swing of the device. Δ*Ψ* is

(5) $$\Delta \Psi =\Psi_{S}^{QM}-\Psi_{S}^{CL},$$

where $\Psi_{S}^{QM}$ and $\Psi_{S}^{CL}$ are the potential at the silicon–oxide interface when considering the quantum models and semi-classical models, respectively.

The Ti-TcFETs have been simulated considering both the Bohm quantum potential (BQP) models and semi-classical models. The threshold voltage of the device is reduced by 0.018 V when considering the BQP quantum compared with semi-classical models. Studies have shown that the amount of threshold voltage drift caused by quantum effects will become obvious when the channel silicon thickness is very thin (<2 nm) [18]. For undoped devices with a bulk silicon thickness (>4 nm), the threshold voltage drift caused by quantum effects is small [18].

### 3.2. Drain Current of the Ti-TcFET

The drain current *I_D_* of the Ti-TcFET device can be expressed by Equation (6).

(6) $$I_{D}=I_{S}\cdot \frac{H_{Fin2}T_{Si2}}{L_{g}}\cdot {\left(V_{top\text{-}gate}-V_{THt}\right)}^{\lambda },$$

where *I_S_* and *λ* are fitting parameters. For short-channel devices, the range of *λ* is about 1.3 to 1.5 [19].

**Figure 4:**
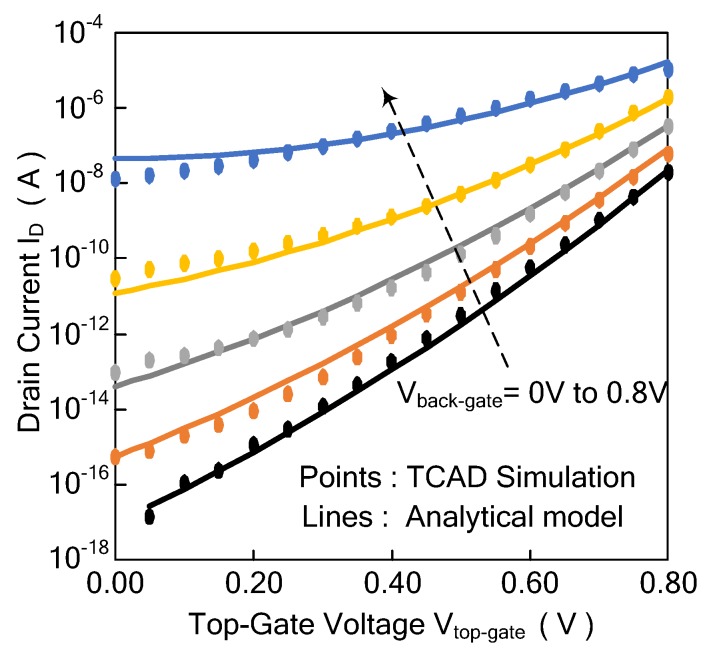
Comparison between the calculated and simulated drain current versus the voltages of the top gate in different back-gate voltages at *V_DS_* = 0.8 V and *V_front-gate_* = 0 V.

The drain current of the Ti-TcFET device versus the voltages of the top gate is shown in Figure 4, where *V_DS_* is 0.8 V, *V_front-gate_* is 0 V, and *V_back-gate_* changes from 0.2 V to 0.8 V. In Figure 4, the points are obtained by the TCAD simulations, while the lines are the theoretical calculation results of the drain current for different voltages of the top gate. The results show that the theoretical formula of the drain current agrees with the TCAD simulation results.

### 3.3. Subthreshold Current of the Ti-TcFET

Referring to the literature [20,21], the subthreshold leakage current *I_sub_* of the Ti-TcFET device can be calculated by Equation (7):

(7) $$I_{sub}=I_{w}\cdot \frac{H_{Fin2}T_{Si2}}{L_{g}}\cdot e^{\frac{\left(V_{top\text{-}gate}-V_{THt}\right)+m\cdot {\left(V_{top\text{-}gate}-V_{THt}\right)}^{2}}{n\cdot v_{T}}}\cdot \left(1-e^{\frac{-q\cdot V_{DS}}{v_{T}}}\right)\cdot e^{b1\cdot V_{top\text{-}gate}+b2\cdot V_{top\text{-}gate}^{2}+b3\cdot V_{top\text{-}gate}\cdot V_{THt}},$$

where *I_w_*, *m*, *b*1, *b*2, and *b*3 are fitting parameters and *n* is subthreshold slope parameter.

**Figure 5:**
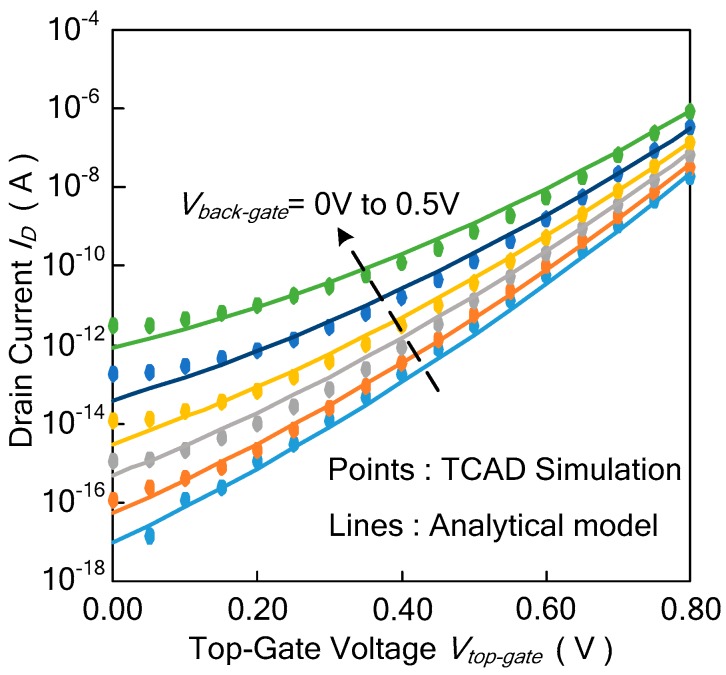
Comparison between the calculated and simulated subthreshold drain current versus the voltages of the top gate in different back-gate voltages at *V_DS_* = 0.8 V and *V_front-gate_* = 0 V.

The drain current of the Ti-TcFET device versus the voltages of the top gate is shown in Figure 5, where *V_DS_* is 0.8 V, *V_front-gate_* is 0 V, and *V_back-gate_* changes from 0 V to 0.5 V. In Figure 5, the points are obtained by TCAD simulations, while the lines are the theoretical calculation results of the drain current for different voltages of the top gate. The calculated subthreshold drains agree well with the simulated subthreshold current for *V_top-gate_* > 0.1 V.

### 3.4. Performance Optimization of the Ti-TcFET Devices

In order to obtain high-performance Ti-TcFET devices, the following two goals should be achieved. If only one gate is activated, the current should be as small as possible. If any two of the three gates are activated, the current should be as large as possible. In other words, the maximum turn-off current *I_off_* should be small and the minimum turn-on current *I_on_* should be large. In this subsection, we study the influence of device size and parameters on device performance by changing the channel thickness, gate oxide thickness, and gate work function, and then select the optimized device size and parameters.

In order to achieve the first goal, Ti-TcFET devices should have a high-threshold voltage when only one gate is activated. The threshold voltage of a Ti-TcFET is approximated by Equation (8):

(8) $$V_{Tht}=V_{inv}+\Phi_{m}+\frac{Q_{D}}{C_{ox}}+V^{QM}-V^{SCE},$$

where *V_inv_* is a constant, *Φ_m_* is the work function difference of the electrode and the silicon*, Q_D_* is the channel depletion charge, *C_ox_* is the oxide capacitance of the front gate, back gate, and top gate, and *V^QM^* and *V^SCE^* are the threshold voltage increase caused by quantum-mechanical effect models and short-channel effects, respectively. From Equation (8), the threshold voltage of the device can be adjusted by selecting a suitable *Φ_m_* and *C_ox_*.

In order to achieve the second goal, Ti-TcFET devices should have a low subthreshold slope, so that the device achieves a large turn-on current with a small turn-off current. The subthreshold slope *S* is given by Equation (9) [22]:

(9) $$S=\frac{\partial V_{top\text{-}gate}}{\partial \log I_{D}}=\ln10\cdot \frac{kT}{q}\cdot \frac{\Delta V_{top\text{-}gate}}{\Delta \psi_{Si}}=60\cdot \frac{\Delta V_{top\text{-}gate}}{\Delta \psi_{Si}},$$

where *ψ_Si_* is the surface potential at the gate electrode. The subthreshold slope can be approximated by Equation (10) [23]:

(10) $$S=60\cdot \frac{T_{oxt}+2.1T_{Si}+T_{oxb}}{T_{oxb}}=60\cdot \left(\frac{2.1T_{Si}}{T_{OX}}+2\right),$$

From Equation (10), we can get the relationship between the subthreshold slope *S*, channel thickness *T_Si_*, and gate oxide thickness *T_ox_*, which can be used to optimize the performances of Ti-TcFET devices.

#### 3.4.1. Effect of Channel Thickness on Current Characteristics

**Figure 6:**
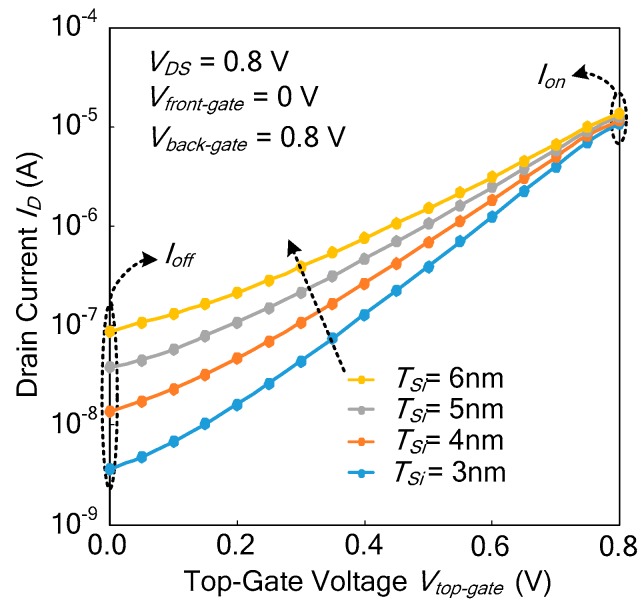
The turn-on current *I_on_* and turn-off current *I_off_* at different channel thicknesses *T_Si_*.

Figure 6 shows the effect of channel thickness *T_si_* on current characteristics at *V_front-gate_* = 0 V and *V_back-gate_* = 0.8 V. When the voltage *V_top-gate_* of the top gate is set as 0.8 V, the two inputs of the N-type Ti-TcFET are at 0.8 V, and thus the device should be turned on. Its drain current is named as *I_on_* (turn-on current). When the voltage of the top gate *V_top-gate_* is 0 V, only one input of the Ti-TcFET is at 0.8 V, and thus the device should be turned off. Its drain current is named as *I_off_* (turn-off current).

From Figure 6, it ca be seen that as the channel thickness reduces from 6 nm down to 3 nm, *I_on_*/*I_off_* (the switching current ratio) increases. From Equation (10), the subthreshold slope *S* of the devices reduces when the channel thickness *T_Si_* reduces. The results show that the TCAD simulations agree with the theoretical formula. In order to have enough *I_on_* and an acceptable *I_on_*/*I_off_*, the optimized *T_Si_* is set to 4 nm.

#### 3.4.2. Effect of Gate Oxide Thickness on Current Characteristics

**Figure 7:**
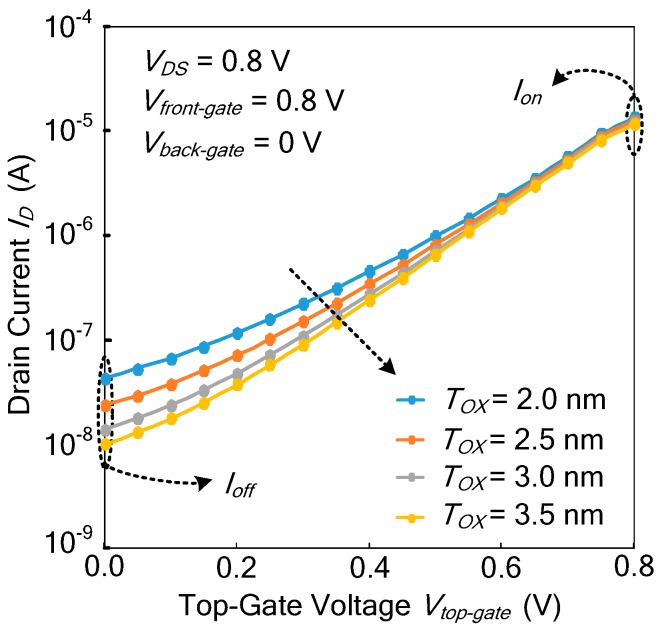
The turn-on current *I_on_* and turn-off current *I_off_* at different thickness *T_OX_* of the dielectric HfO_2_.

Figure 7 shows the effect of gate oxide thickness *T_OX_* on current characteristics at *V_front-gate_* = 0 V and *V_back-gate_* = 0.8 V according to the TCAD simulations. As the gate oxide thickness *T_OX_* increases from 2.0 nm to 3.5 nm, *I_on_*/*I_off_* increases. As shown in Equation (10), when the gate oxide thickness *T_OX_* increases, the subthreshold slope *S* of the Ti-TcFET devices decreases, so that *I_on_*/*I_off_* increases. The results show that the theoretical formula agrees with the TCAD simulations. In order to have enough *I_on_* and an acceptable *I_on_*/*I_off_*, the optimized gate oxide thickness *T_OX_* is set to 3 nm.

#### 3.4.3. Effect of Gate Work Function on Current Characteristics

**Figure 8:**
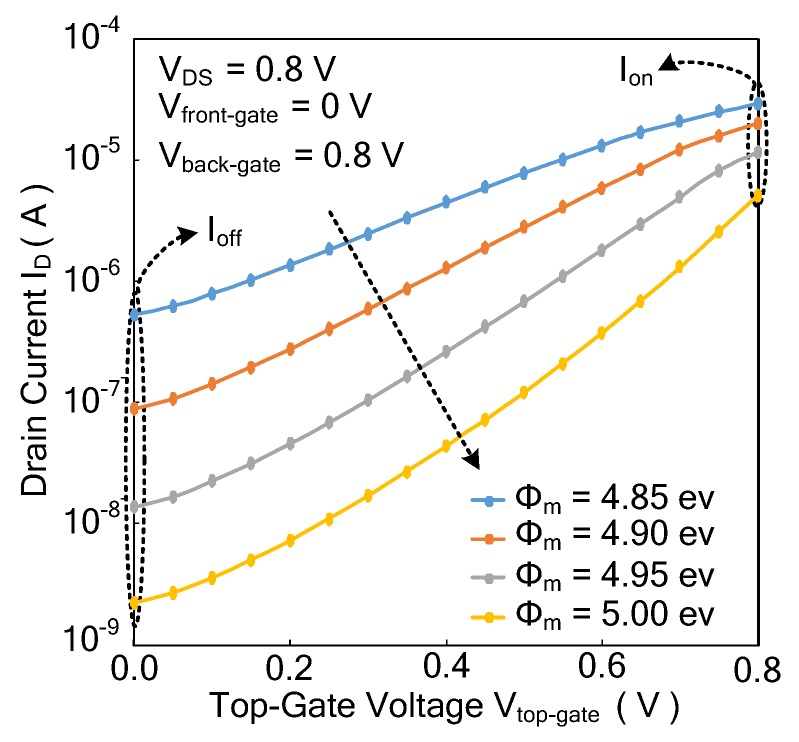
The turn-on current *I_on_* and turn-off current *I_off_* with different gate work functions *Φ_m._*

Figure 8 shows the effect of the gate work function on current characteristics at *V_front-gate_* = 0 V and *V_back-gate_* = 0.8 V according to the TCAD simulations. From Figure 8, it can be seen that as the gate work function *Φ_m_* increases from 4.85 eV to 5.00 eV, *I_off_* decreases. As shown in Equation (8), a high-threshold voltage can be achieved by increasing the gate work function *Φ_m_*, so that *I_off_* decreases. The results show that the theoretical formula agrees with the TCAD simulations. In order to reduce *I_off_*, the optimized gate work function is set to 4.95 eV.

### 3.5. Drain-Induced Barrier Lowering (DIBL) and S of the Optimized Ti-TcFET Devices

The drain-induced barrier lowering (DIBL) can be calculated by using Equation (11):

(11) $$DIBL(mV/V)=\frac{\Delta V_{Tht}}{\Delta V_{DS}},$$

**Figure 9:**
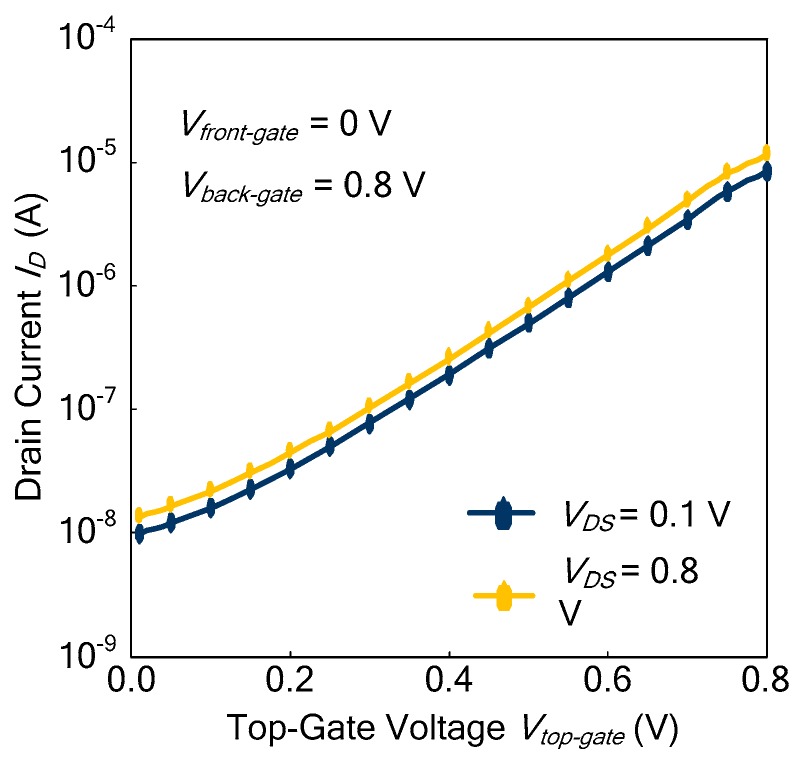
Drain current of the Ti-TcFET when *V_front-gate_* = 0 V and *V_back-gate_* = 0.8 V.

As shown in Equation (11), the DIBL is defined as the difference in threshold voltage when the drain voltage is increased. The drain current of the Ti-TcFET is shown in Figure 9 when *V_front-gate_* = 0 V and *V_back-gate_* = 0.8 V. From Figure 9, the DIBL of the Ti-TcFET device is about 41.48 mV/V when *V_front-gate_* = 0 V and *V_back-gate_* = 0.8 V. Our TCAD simulations show that the DIBL of the Ti-TcFET device is almost the same as the standard FinFET device when using the same device parameters.

**Figure 10:**
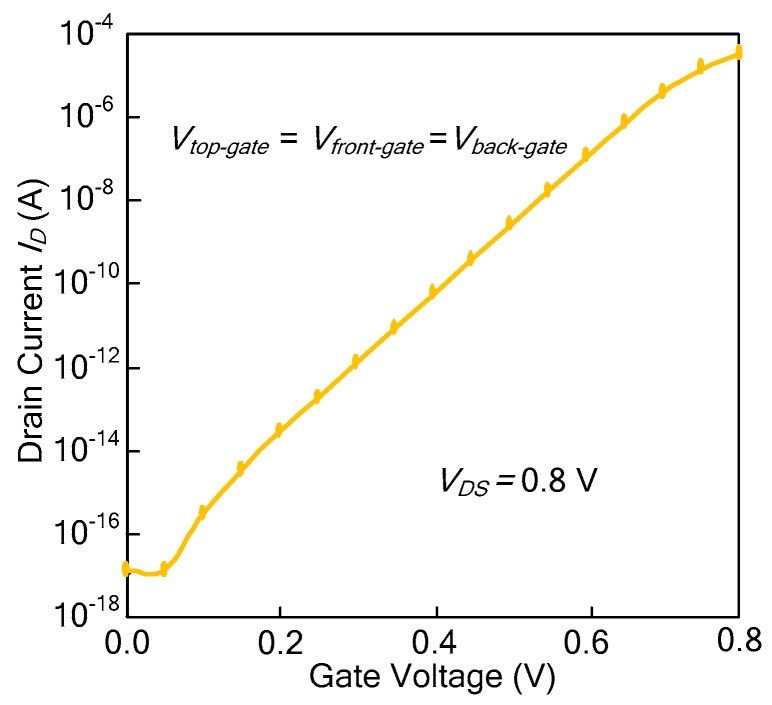
Drain current of the Ti-TcFET when *V_top__-gate_* = *V_front-gate_* = *V_back-gate_*.

The drain current of the Ti-TcFET is shown in Figure 10 when *V_top-gate_* = *V_front-gate_* = *V_back-gate_*. From Figure 10, it can be seen that the subthreshold slope *S* of the Ti-TcFET devices is about 62.6 mV/dec. The TCAD simulations show that the subthreshold slope *S* of the Ti-TcFET device is also almost the same as the standard FinFET device when using the same device parameters.

### 3.6. Scaling Factors of the Ti-TcFET Devices

**Figure 11:**
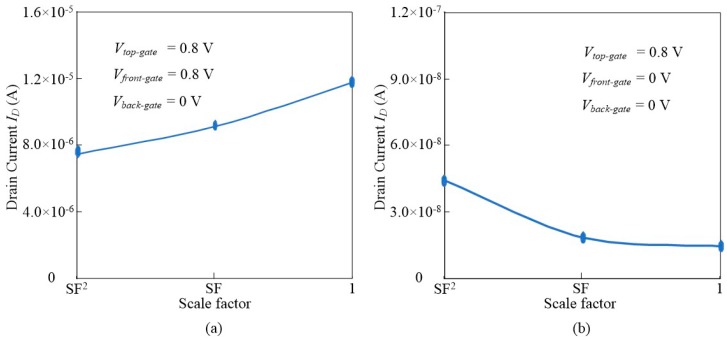
(**a**) The minimum turn-on current as channel length *L_g_* and fin height *H_Fin_* scale down, and (**b**) the maximum turn-off current as channel length *L_g_* and fin height *H_Fin_* scale down. The scaling factor (SF) is set as 0.707.

The minimum turn-on current and maximum turn-off current are shown in Figure 11 as channel length *L_g_* and fin height *H_Fin_* scale down, where the scaling factor (SF) is set as 0.707. From Figure 11a, as the channel length *L_g_* and fin height *H_Fin_* scale down, the minimum turn-on current reduces slowly. From Figure 11b, as the channel length *L_g_* and fin height *H_Fin_* scale down, the maximum turn-off drain is almost a constant.

### 3.7. Performance Analysis of the Ti-TcFET Devices

**Figure 12:**
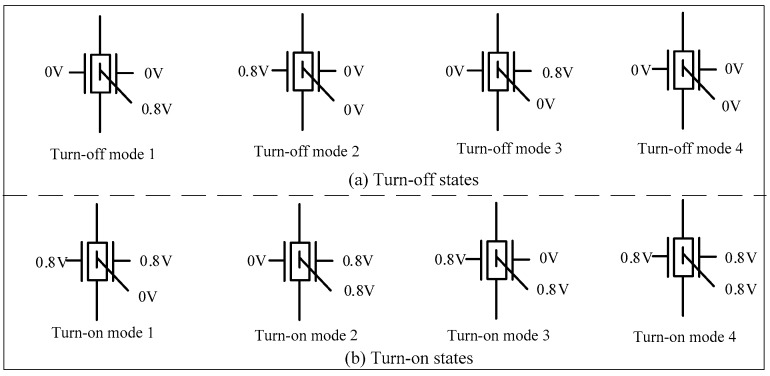
Eight switching modes of the Ti-TcFET device.

The Ti-TcFET device has three input terminals, and each input terminal has two logic values with logic “1” (0.8 V) and logic “0” (0 V), so that the device has eight switching modes. Taking the N-type Ti-TcFET as an example, eight working modes are illustrated in Figure 12. When only one or fewer inputs are “1”, the device is turned off, as shown in Figure 12a. In Figure 12a, there are four switching modes in the turn-off state. When two or three inputs are “1”, the device is turned on, as shown in Figure 12b. In Figure 12b, there are also four switching modes in the turn-on state.

Using the optimized parameters and device sizes listed in Table 1, the turn-on and turn-off currents of N-type and P-type Ti-TcFETs working in eight switching modes are listed in Table 2 and Table 3, respectively.

Table 2 lists the turn-on current *I_on_* and turn-off current *I_off_* of the N-type Ti-TcFET in the four turn-on modes and four turn-off modes, respectively. The normalized currents are listed in the rightmost column in Table 2. From Table 2, it can be seen that the maximum turn-off current among the four turn-off modes is 2.06 × 10^−8^ A, while the minimum turn-on current among the four turn-on modes is 1.14 × 10^−5^ A. In the worst case, *I_on_*/*I_off_* (switching current ratio) is 553. In order to increase *I_on_*/*I_off_*, some new materials such as ferroelectric materials and two-dimensional materials can be used. Ferroelectric materials can enhance the internal gate voltage through the negative capacitance effect, so that *I_on_*/*I_off_* (switching current ratio) increases greatly and the subthreshold swing decreases below 60 mV/dec [24]. Two-dimensional (2D) semiconductors, such as transition metal dichalcogenides (TMDs), have the potential for ultra-scaled transistor technology beyond 10 nm node technology because of their atomically thin layered channel and low dielectric constant, which offer strong electrostatic control [25].

Table 3 shows the turn-on current *I_on_* and turn-off current *I_off_* of the P-type Ti-TcFET in the four turn-on modes and four turn-off modes, respectively. The normalized currents are also listed in the rightmost column in Table 3. From Table 3, it can be seen that the maximum turn-off current among the four turn-off modes is 1.57 × 10^−8^ A, while the minimum turn-on current among the four turn-on modes is 7.09 × 10^−6^ A. In the worst case, the switching current ratio is 452. 

### 3.8. Logic Cells Based on Ti-TcFET Devices

**Figure 13:**
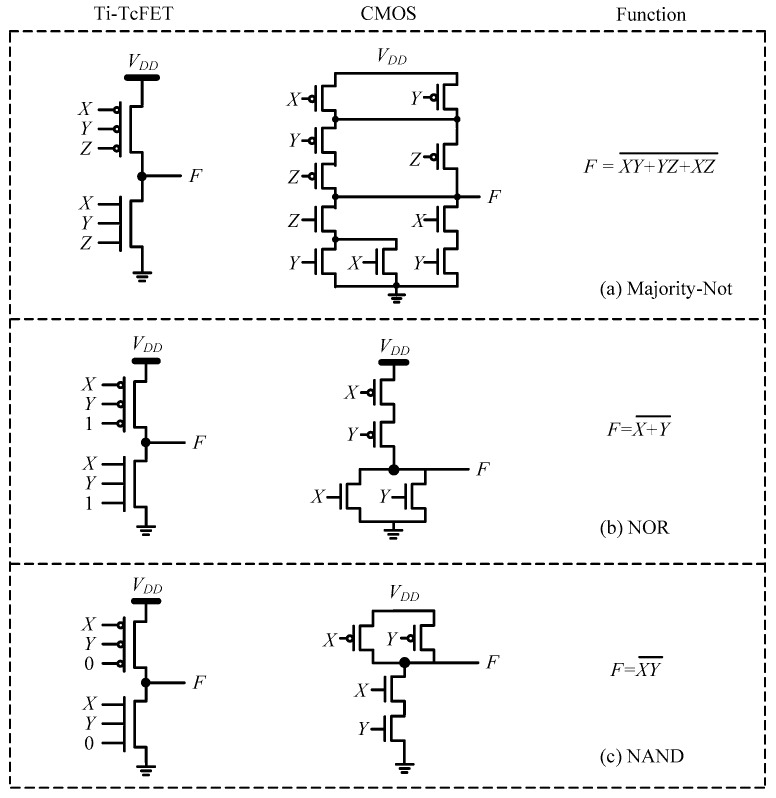
The logic cells based on Ti-TcFET devices: (**a**) majority-not, (**b**) NOT-OR (NOR), and (**c**) NOT-AND (NAND).

A single Ti-TcFET transistor can implement three input “majority-not” switch functions [26,27], and thus only one N-type Ti-TcFET and one P-type Ti-TcFET are needed to realize a “majority-not” logic cell, as shown in Figure 13a. From Figure 13a, the “majority-not” logic cell using traditional CMOS/FinFET devices needs 10 transistors. The transistor counts of the “majority-not” cell using the proposed Ti-TcFET devices is only one-fifth of that of the “majority-not” cell using traditional CMOS/FinFET devices, which shows that the proposed Ti-TcFET devices with three input terminals have a higher information processing capacity than traditional CMOS/FinFETs with a single input terminal.

**Figure 14:**
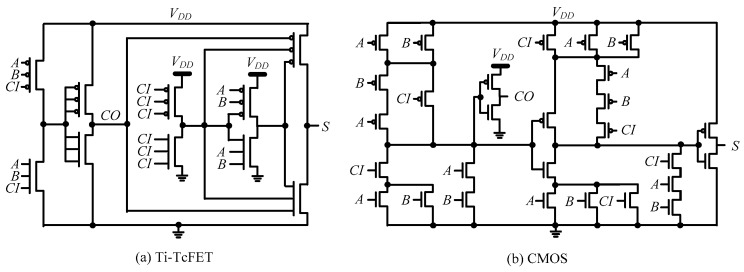
Full adders based on Ti-TcFET devices (**a**) and complementary metal–oxide–semiconductor (CMOS) devices (**b**).

The Ti-TcFET devices can also be used to implement other logic gates in a compact style, such as NOR and NAND, as shown in Figure 13b,c, respectively. For more complex logic circuits, such as a full adder, the circuit structure can also be simplified by using Ti-TcFET devices, as shown in Figure 14a. For comparison, Figure 14b shows the full adder using traditional CMOS/FinFETs.

The power consumption, delay, and power delay product of the one-bit full adder using Ti-TcFET devices and standard FinFET devices are compared in Table 4. From Table 4, the power consumption and power delay product of the one-bit full adder based on Ti-TcFET devices are smaller than standard FinFET devices, with an acceptable delay penalty.

## 4. Conclusions

In this paper, a novel T-channel field effect transistor with three input terminals (Ti-TcFET) is proposed. The T-channel structure increases the coupling area between the top gate and the front and back gates so that the device can realize the “majority-not” function well. By adjusting the gate work function, channel thickness, and the thickness of the gate oxide layer, the performance of the Ti-TcFET device is optimized. The results show that when the gate work function *Φ* of the N-type Ti-TcFET is 4.95 eV, *T_Si_* = 4 nm, and *T_OX_* = 3 nm, the minimum turn-on current *I_on_* is 1.14 × 10^−5^ A and the maximum turn-off *I_off_* is 2.06 × 10^−8^ A, with the switching current ratio *I_on_*/*I_off_* of 553. When the gate work function *Φ* of the P-type Ti-TcFET is 4.52 eV, *T_Si_* = 4 nm, and *T_OX_* = 3 nm, the minimum turn-on current *I_on_* is 7.09 × 10^−6^ A and the maximum turn-off current *I_off_* is 1.57 × 10^−8^ A, with the switching current ratio *I_on_*/*I_off_* of 452. The purpose of this paper was to propose a three input device to simplify the circuit structure and thus to provide a new idea for future circuit designs.

In the future, we will optimize the N-type and P-type Ti-TcFETs by applying new materials such as ferroelectric materials and two-dimensional materials, which should be helpful to increase the switching current ratio of the device and to decrease the leakage current.

## Figures and Tables

**Table 1:** Parameters of the T-channel field effect transistor with three input terminals (Ti-TcFET) device.

Parameter	Optimized Value	Parameter	Optimized Value
Gate dielectric thickness (*T_ox_*)	3 nm	Drain doping (*N*_drain_)	2 × 10^20^ cm^−3^
Channel thickness 1 (*T_Si1_*)	4 nm	Source doping (*N*_source_)	2 × 10^20^ cm^−3^
Channel thickness 2 (*T_Si2_*)	4 nm	Channel doping (*N*_channel_)	1 × 10^16^ cm^−3^
Fin height 1 (*H_Fin1_*)	40 nm	Gate work function (*Φ_m_*)	4.52 eV (P-type)
Fin height 2 (*H_Fin2_*)	84 nm		4.95 eV (N-type)
Gate length (*L_g_*)	24 nm	-	-

**Table 2:** The turn-on current and turn-off currents of the N-type Ti-TcFET.

State	*V_top-gate_*, *V_front-gate_*, *V_back-gate_*	*I_D_* (A)	Normalized
Turn-off mode 1	0.8 V, 0 V, 0 V	2.06 × 10^−8^	0.0005
Turn-off mode 2	0 V, 0.8 V, 0 V	1.40 × 10^−8^	0.0004
Turn-off mode 3	0 V, 0 V, 0.8 V	1.40 × 10^−8^	0.0004
Turn-off mode 4	0 V, 0 V, 0 V	1.47 × 10^−17^	3.81 × 10^−13^
Turn-on mode 1	0 V, 0.8 V, 0.8 V	1.17 × 10^−5^	0.3031
Turn-on mode 2	0.8 V, 0 V, 0.8 V	1.17 × 10^−5^	0.3031
Turn-on mode 3	0.8 V, 0.8 V, 0 V	1.14 × 10^−5^	0.2953
Turn-on mode 4	0.8 V, 0.8 V, 0.8 V	3.86 × 10^−5^	1.0000

**Table 3:** The turn-on current and turn-off currents of the P-type Ti-TcFET.

State	*V_top-gate_*, *V_front-gate_*, *V_back-gate_*	*I_D_* (A)	Normalized
Turn-off mode 1	0 V, 0.8 V, 0.8 V	1.57 × 10^−8^	0.0007
Turn-off mode 2	0.8 V, 0 V, 0.8 V	1.06 × 10^−8^	0.0004
Turn-off mode 3	0.8 V, 0.8 V, 0 V	1.06 × 10^−8^	0.0004
Turn-off mode 4	0.8 V, 0.8 V, 0.8 V	2.75 × 10^−17^	1.15 × 10^−12^
Turn-on mode 1	0.8 V, 0 V, 0 V	7.48 × 10^−6^	0.3116
Turn-on mode 2	0 V, 0.8 V, 0 V	7.09 × 10^−6^	0.2954
Turn-on mode 3	0 V, 0 V, 0.8 V	7.09 × 10^−6^	0.2954
Turn-on mode 4	0 V, 0 V, 0 V	2.40 × 10^−6^	1.0000

**Table 4:** The power consumption, delay, and power delay product of the one-bit full adder using Ti-TcFET devices and standard FinFET devices.

Full Adder	Power Consumption (nW)	Delay (pS)	Power Delay Product (zJ)
FinFET	19.29	44.38	856
Ti-TcFET	9.26	59.59	554
